# Measures assessing attributes of evidence-informed decision-making (EIDM) competence among nurses: a systematic review protocol

**DOI:** 10.1186/s13643-018-0849-8

**Published:** 2018-11-03

**Authors:** Emily Belita, Jennifer Yost, Janet E. Squires, Rebecca Ganann, Trish Burnett, Maureen Dobbins

**Affiliations:** 1 School of Nursing, McMaster University, McMaster Innovation Park (MIP), 175 Longwood Road South, Suite 210a, Hamilton, ON L8P 0A1 Canada; 2 M. Louise Fitzpatrick College of Nursing, Villanova University, Villanova, PA USA; 3 School of Nursing/École des sciences infirmières, University of Ottawa/Université d’Ottawa, Ottawa, ON Canada; 4 National Collaborating Centre for Methods and Tools, Hamilton, ON Canada

**Keywords:** Evidence-informed decision-making, Competence, Assessment, Psychometrics, Nursing

## Abstract

**Background:**

There are growing professional expectations for nurses to engage in and develop competence in evidence-informed decision-making (EIDM) due to opportunities for improved client and community outcomes and provision of the highest quality of care. However, EIDM is underdeveloped, with low implementation rates among nurses. The use of indicators to assess EIDM performance has potential to encourage nurses’ engagement in EIDM through competence recognition and support assessment of strengths and competency gaps for individual nurses and organizations. Currently, the state of evidence regarding measures that assess EIDM competence attributes (i.e., knowledge, skills, beliefs/values, behaviors) among nurses is unknown. This systematic review aims to address this gap through a narrative synthesis of the characteristics and psychometric properties of EIDM competence measures.

**Methods:**

The search strategy, developed in consultation with a Health Sciences Librarian, consists of online databases, contacting experts, hand searching reference lists, key journals, websites, conference proceedings, and grey literature. Studies will be included if the following criteria are met: (1) sample includes practicing nurses and data for nurses are reported separately; (2) conducted in any healthcare setting; (3) quantitative or mixed-methods design; (4) reports use or testing of a measure assessing EIDM competence attributes (i.e., knowledge, skills, attitudes/values, and/or behaviors); and (5) published in English. Screening will be conducted independently by two reviewers using a two-stage process: (1) title and abstract level; and (2) full-text level. Data extraction of study characteristics (e.g., sample, setting) will be conducted by a single reviewer and checked for accuracy by a second reviewer. Psychometric properties of acceptability, reliability, and validity evidence for each measure will be independently extracted by two reviewers. Data on measures will be synthesized narratively according to acceptability, number of validity evidence sources established, and reliability of scores. Data pertaining to population and healthcare setting will also be reported for each measure.

**Discussion:**

This systematic review will provide a current understanding about the state of evidence with respect to EIDM competence measures in nursing to assist in determining potentially relevant and robust measures for use in different nursing practice settings.

**Systematic review registration:**

Protocol registered in PROSPERO Registration #: CRD42018088754

**Electronic supplementary material:**

The online version of this article (10.1186/s13643-018-0849-8) contains supplementary material, which is available to authorized users.

## Background

Evidence-informed decision-making (EIDM) is defined as a process in which high quality, available evidence from research, local data, patient and professional experiences are synthesized, disseminated, and applied to decision-making in healthcare practice and policy [1, 2]. Considerable attention to the integration of EIDM in clinical practice is warranted given substantial benefits to the healthcare system, healthcare professionals, clients, and communities. The most critical reason for implementing EIDM is the potential of providing the highest quality of care due to the use of more effective and cost-efficient interventions, resulting in the best client outcomes across healthcare settings [3, 4]. Examples of improved client care and outcomes following EIDM implementation are best demonstrated in knowledge translation studies that support integration of research evidence in practice. In a systematic review of 10 knowledge translation studies focused on mentoring, Abdullah and colleagues [5] report outcomes of improved physician prescribing behaviors for acute myocardial infarction and improved documentation of pain assessments by nurses following increased uptake of best practice clinical guidelines. Yost et al. [6] also conducted a systematic review of 30 studies with an aim to determine the effect of knowledge translation interventions on client outcomes. Across these studies, diverse client outcomes are reported including a reduction in patient falls, reductions in pain intensity following uptake of guidelines for pain assessment and management in older adults, a clinically significant reduction in death risk and dependency, and improved physical health after implementation of best practice guidelines for acute stroke care.

In considering benefits for nurses, EIDM also promotes empowerment and job satisfaction [7, 8], facilitates professional development and advances the nursing profession [9], and may support nursing retention [10].

The importance of EIDM is further underscored by its inclusion in national practice frameworks and provincial practice standards for nurses. In the Framework for the Practice of Registered Nurses in Canada [11], the Canadian Nurses Association identifies EIDM as a fundamental principle to nurses’ development of clinical expertise and maintenance of overall competence. In a position statement on EIDM and nursing practice, the Canadian Nurses Association establishes professional responsibilities related to EIDM for various nursing roles including frontline clinicians, educators, researchers, nursing regulatory bodies, and professional associations [2]. The professional expectation is that nurses develop the necessary competencies to engage in EIDM which include, but are not limited to, reading and critically appraising scientific literature, identifying and articulating clinical research questions for investigation, as well as supporting the evaluation and promotion of EIDM [2]. EIDM competency expectations are emphasized even further in specific nursing practice settings. For example, the Community Health Nurses of Canada [12] has identified critical appraisal and use of evidence in the development of public health policies and practice as core competencies for all nurses practicing in a community setting. Provincially, the College of Nurses of Ontario [13] established professional practice standards with respect to accountability, knowledge, and knowledge application. Within these, indicators demonstrating achievement of standards describe nurses’ abilities to develop evidence-informed rationale for interventions and apply evidence in every day practice [13].

Despite professional expectations to engage in EIDM and benefits to the healthcare system, professionals, and clients, there remain critical deficits in EIDM implementation and competence. Across diverse and international nursing practice settings, EIDM implementation has been primarily deemed low [14–17] and at its very best, moderate [18]. In a recent integrative review [18], studies reported many shortfalls among nurses with respect to EIDM competence attributes of knowledge, skills, and behaviors. EIDM knowledge varied considerably among nurses, and in some cases, there was a discrepancy between nurses’ understanding of EIDM primary concepts and how EIDM is most commonly conceptualized [18]. For example, in international samples of nurses, EIDM was believed to be nursing practice based solely on intuition, tradition, or professional experience without the integration of other forms of evidence such as research or client preferences and values [18]. Across studies, nurses also consistently reported that the EIDM knowledge and skills they possessed were insufficient to engage in EIDM implementation and practice change [18]. Additionally, in a recent cross-sectional study, a large sample of hospital nurses from the USA collectively rated themselves as below ‘competent’ across 24 EIDM competencies [19].

In the nursing literature, barriers hindering EIDM are well documented. Prominent organizational barriers reported by nurses include lack of protected work time for EIDM, lack of strategic vision or leadership in EIDM, and exclusion from clinical decision-making [17, 20, 21]. Related to this is a lack of clarity, consistency, formal processes, and structures to define EIDM roles and expectations [22], as well as lack of specificity in EIDM competencies, preventing organizations from meeting standards of high quality healthcare that is evidence-informed [14, 23]. In contrast, an overarching facilitator of EIDM is a supportive organizational culture that includes nursing leaders championing EIDM work through mentorship and participation in strategic visioning to support frontline EIDM uptake [20, 24]. Proposed organizational strategies to encourage EIDM implementation include the explicit addition of EIDM indicators to appraisal processes for practitioners [22], development of EIDM practice standards [25], and use of clear EIDM competencies specific to general class nurses and those in advanced practice [23]. Establishing clarity, consistency, and a rigorous assessment method for EIDM competence provides clear direction for knowledge and skill development, in addition to competence recognition providing further motivation for EIDM engagement [26].

Given this, competence assessment serves a critical role in sustaining and improving EIDM implementation among nurses. The focus on assessment of competence and continuing competence (i.e., maintenance and continual improvement of competence) is supported by the College of Nurses of Ontario to ensure high quality patient care and promote the advancement of nursing and continued professional learning [27]. The College of Nurses of Ontario [13] asserts that “competence is the nurse’s ability to use her/his knowledge, skill, judgment, attitudes, values and beliefs to perform in a given role, situation and practice setting” (p. 5). More specifically, competence is conceived of as the amalgamation of attributes including knowledge, skills, attitudes/values, and behaviors applied to performance [28–30].

Critical attributes of EIDM competence are articulated across the literature. EIDM knowledge refers to an understanding about the defining theoretical, practical concepts and principles of EIDM, and the different levels of evidence [31–35], whereas EIDM skills are universally understood as the application of such knowledge to perform EIDM tasks [31–35]. Tilson et al. [33] and Buchanan et al. [31] offer the most developed definition of attitudes/values, which are described as perceptions, personal beliefs about, and the importance assigned to EIDM. This includes believing that EIDM is associated with positive outcomes and valuing each separate step of the EIDM process [33]. The enactment of EIDM steps in a real-world clinical setting (e.g., searching databases for evidence, accessing information sources) define the competence attribute of behavior [31, 33, 35].

Measures assessing individual EIDM competence attributes separately exist in different healthcare disciplines including nursing [36], allied healthcare [37, 38], and medicine [39, 40]. To date, there is only one systematic review [32] that identifies measures to assess nurses’ and midwives’ knowledge, skills, and attitudes for EIDM. However, while the intent of the review was to focus on a population of nurses and midwives in clinical environments, its included studies, to some extent, did include samples of medical practitioners and allied health professionals. Leung et al. acknowledge different EIDM competence attributes such as knowledge, skills, and attitudes [32]; missing from this, however, is the competence attribute of ‘behavior’ with the consequence of potentially excluding such critical measures in the review.

Also noteworthy is the inclusion of research utilization measures in the review by Leung et al. [32]. Conceptually, the difference between EIDM and research utilization is well articulated in the literature [41, 42]. Research utilization encompasses a component of, and is housed under the broader definition of EIDM [41–43]. The critical difference between these concepts involves the form of evidence applied to healthcare practice. Research utilization emphasizes the use of research evidence that is scientific/empirical in nature [41]. While, EIDM denotes use of a broader understanding of evidence that includes integration of not only research, but also evidence from clinical experience, clients and caregivers, and local context or environment [44]. Despite this conceptual difference, measures originally developed to assess research utilization were still included in the review if their use in subsequent studies was cited as measuring EIDM. Data extraction also did not include healthcare setting (e.g., acute care, community), which would make it difficult to determine a measure’s relevance for use in a specific nursing setting. Coupled with this, assessment of validity evidence was guided by the traditional Trinitarian approach of treating criterion, content, and construct validity as separate entities [45], rather than using the contemporary approach of understanding validity evidence as a unified concept according to the Standards for Educational and Psychological Testing [46]. Not having a comprehensive assessment of validity evidence for a measure in relation to a specific population and healthcare setting would make it difficult to determine appropriateness given a particular context.

As such, the proposed systematic review aims to address these limitations within the existing literature and contribute to a current understanding about the state of evidence in EIDM competence assessment in nursing.

### Objectives

The objectives of this systematic review are to (1) comprehensively identify existing measures of EIDM competence attributes (i.e., knowledge, skills, attitudes/values, and/or behaviors) used among nurses; and (2) assess and synthesize their psychometric properties (i.e., acceptability, reliability, validity). The contemporary understanding of psychometric assessment will be guided by the Standards for Educational and Psychological Testing [46]. Narrative synthesis of this data, coupled with identification of practice settings and sample population, will provide a current understanding of existing EIDM competence measures to assist healthcare institutions in determining relevant and robust measures for use in specific nursing practice settings.

## Methods

This protocol has been registered a priori in PROSPERO (#CRD42018088754) and follows the Preferred Reporting Items for Systematic Reviews and Meta-Analyses (PRISMA) guidelines as included in Additional file 1 [47].

### Search strategy

A comprehensive search strategy was developed in consultation with a Health Sciences Librarian. The primary online databases to be searched include Ovid MEDLINE, EMBASE, CINAHL, and ERIC. Statistical databases (Health and Psychosocial Instruments [HaPI] and MathSciNet) will also be searched as they index primarily psychometric studies. Search terms will differ according to unique subject headings in each database. An example of a search strategy for MEDLINE is available in Additional file 2. Date limitations will be from 1990 until the current date. The year limit of 1990 was selected as this was when the concept of evidence-based medicine was officially coined with further development of the construct’s conceptual nature occurring throughout the 1990s. Reference lists of included studies, highly relevant journals (Implementation Science, Worldviews on Evidence Based Nursing), and conference abstracts and/or proceedings of highly relevant conferences (Annual Conference on the Science of Dissemination and Implementation in Health, Community Health Nurses of Canada Conference, Knowledge Translation Canada Annual Scientific Meeting) will be hand searched. Strategies for locating gray literature will include contacting experts in the field of EIDM competence assessment; searching gray literature databases including ProQuest Dissertations and Theses, Greylit.org, Canadian Health Research Collection; and using a targeted approach of searching publication portals on websites of the Canadian Nurses Association, Community Health Nurses Association of Canada, and American Nurses Association for assessment tools. Duplicates will be removed and all unique references will be screened for relevance.

### Inclusion criteria

Studies will be included if they meet the following criteria: (1) study sample consists entirely of nurses or a portion of the sample comprises nurses for which data is presented separately or can be extracted; (2) take place in any healthcare setting (e.g., public health, hospital, primary care, long-term care); (3) report results from testing or the use of a measure that assesses any EIDM competence attribute (i.e., knowledge, skills, attitudes/values, behaviors). (See Additional file 3 for attribute definitions); (4) includes measures with a quantitative design or mixed-methods design; and (5) written in English.

### Exclusion criteria

Studies will be excluded based on the following criteria: (1) they include measures of EIDM competence used among healthcare professionals other than nurses and nursing specific data is not reported separately or cannot be extracted; (2) the full sample or a portion consists of undergraduate nursing students and data for practicing nurses is not reported separately or cannot be extracted; and (3) measures that solely evaluate research utilization (defined as only one component of EIDM).

### Study selection

Two independent reviewers will screen references at the title and abstract level using the aforementioned inclusion criteria. Citations will be classified into three groups labeled “include,” “exclude,” or “unsure.” Those classified under “include” or “unsure” by either reviewer will move forward into the next round of full-text review. From these results, full-text articles will be assessed independently by two reviewers using more detailed screening inclusion criteria. Screening criteria at the title and abstract level, as well as full-text review is provided in Additional file 4. Citations will be classified in groups for “inclusion” or “exclusion.” Consensus will be used to resolve disagreement at this stage of full-text review. If consensus cannot be met, a third team member will serve as an arbitrator to decide on final inclusion or exclusion. The number of studies identified from information sources, screened for eligibility, included in the review, and excluded studies with reasons identified will be presented in a flow chart using PRISMA guidelines [47]. DistillerSR will be used to screen citations, upload references, and document reasons for study inclusion or exclusion.

### Data extraction

Data extraction will be conducted using a predetermined online data extraction form. The form will be piloted independently by two reviewers on five randomly selected references, discussed, and revised as needed following the pilot. One reviewer will independently extract data pertaining to study characteristics. Thereafter, a second reviewer will check study characteristic data for accuracy. Study characteristics include study design, sample size, professional designation of sample, healthcare setting, geographic location of study, funding, name of measure, format, purpose of measure, item development process, number of items, theoretical framework used, conceptual definitions established, EIDM attributes measured, EIDM domains/steps covered, and description of marking key or scale for self-report measures.

Two reviewers will independently extract data relating to the primary outcomes consisting of the psychometric properties of measures, which include evidence for acceptability, reliability, and validity. Acceptability refers to how acceptable it is for an individual to complete an instrument and will be assessed by extracting data on the proportion of missing responses and time to complete the instrument [48]. Data extraction pertaining to evidence of reliability and validity will be guided by the Standards for Educational and Psychological Testing [46]. Reliability is defined as the consistency of scores from a measure across repeated measurements of different circumstances [46]. Reliability evidence to be extracted may be presented in the form of standard errors of measurement, reliability or generalizability coefficients, or test information functions based on item response theory [46]. Validity is defined as “the degree to which evidence and theory support the interpretations of test scores for proposed uses of tests” (p. 11) [46]. As such, a measure cannot be identified as being valid or not valid, rather, validity is a property of the interpretation of test scores [49]. Producing a strong validity argument to support the interpretation of test scores requires an accrual of various sources of validity evidence [46, 49]. Therefore, data extraction as it relates to validity will focus on different types of evidence based on test content, response processes, internal structure, and relations to other variables. To assist with extracting data from study results that support validity evidence based on relations to other variables, tables will be developed with established theoretical and empirical literature that can be used as guidelines. These tables will be used as guides to determine support for or against validity evidence for a particular measure in which agreement by both data extractors on these decisions will be required. Any discrepancies in data collection will be resolved by consensus between the two reviewers. If during extraction information is incomplete or missing, attempts will be made by one reviewer to contact publication authors and obtain further information. If consensus is not achieved, a third team member will serve as arbitrator for final decisions. DistillerSR will be used by reviewers to document data extraction.

### Data synthesis

Evidence of acceptability, reliability, and validity will be presented narratively. A summary of acceptability findings will be reported for each separate measure. Reliability findings will also be reported for scores of each measure according to the different categories (i.e., standard error measurement, reliability/generalizability coefficients, test information functions) outlined in the Standards for Psychological and Educational Testing [46]. In synthesizing validity evidence data, other reviews have developed their own system of classifying measures according to various levels [35, 50] or by assigning scores [51, 52] based on the number of validity evidence sources for scores of a particular measure. To follow suit, measures will be categorized into four groups, based on the number of validity evidence sources established across studies (e.g., group 1 = 4 sources of validity evidence established, group 2 = 3 sources of validity evidence). The groupings will help identify the state of validity evidence for scores of each measure, contributing to an understanding about the psychometric performance of measures. Along with psychometric data, study characteristics will also be presented with regard to population and healthcare setting.

## Discussion

While this study features a comprehensive search strategy and rigorous systematic review methodology, the authors acknowledge a limitation with respect to quality assessment of primary studies. Such assessment has varied widely across previous psychometric systematic reviews. Commonly, modified versions or components of the original COnsensus-based Standards for the selection of health Measurement INstruments (COSMIN) [53] have been used to critically appraise single studies in systematic reviews focused on measuring EIDM among allied healthcare professionals [31, 54]. An updated COSMIN risk of bias tool was developed [55] and applied in a recent systematic review of self-report measures for alcohol consumption [56]. However, limitations in using the COSMIN in the above cases exist. Leung et al. [32] note that the intended use of the COSMIN is for patient-reported outcome measures, and the use of such out of this context potentially skews overall study quality assessment due to some irrelevant criteria. This is an important consideration as the context of patient-reported outcome measures differs critically from that of the proposed study focused on measures of EIDM competence among healthcare professionals (i.e., nurses). McKenna et al. [56] also identify a limitation with the updated COSMIN tool using a ‘lowest score counts’ method in different categories, as one low score heavily influences overall quality assessment despite high ratings on other assessment criteria.

Other methodological quality assessment criteria such as the CanChild Outcome Measures Guidelines [57] have also been used in previous reviews [34]. Although, its original purpose was to support rating the adequacy of childhood disability measures. Testing for evidence of validity or reliability has also not been reported with respect to this tool. While, critical appraisal in the review by Leung et al. [32] was conducted using their self-developed Psychometric Grading Framework [58] which measures the strength or quality of an instrument based on reliability and validity outcomes, rather than on study methodology.

Across all existing measures (i.e., COSMIN, CanChild Outcome Measures Guidelines, Psychometric Grading Framework) intended to assess study or instrument quality in the context of a psychometric systematic review, there is one common limitation. All measures employ a Trinitarian understanding of validity and assess quality of the study or measure only as it pertains to criterion, construct, and content validity [45]. This proposed study, however, is guided by a modern perspective of validity established in the Standards for Education and Psychological Testing [46] which posits that validity is a unified concept, and all types of validity evidence (i.e., content, internal structure, response process, relationships to other variables) contribute equally to a validity argument [45]. This approach requires an assessment of all evidence across studies to rate validity, and such cannot be determined until all data on validity evidence is extracted and synthesized. Given this, methodological quality assessment after data extraction is not appropriate. As such, this review instead, will focus on the synthesis of validity evidence in reference to the strength of a validity argument using a classification approach based on the number of validity evidence sources established similarly applied in other reviews [35, 50].

## Conclusions

Despite limitations of the proposed study, there is critical contribution to the field of EIDM in nursing practice. The narrative synthesis resulting from this review will present important data on measure characteristics, such as population, healthcare setting, and EIDM competence attributes, in addition to the psychometric properties of validity evidence, reliability, and acceptability; this is largely missing from previous psychometric systematic reviews on EIDM measurement. The comprehensiveness of this synthesis facilitates easy selection or determination of relevance for nursing leaders or individual nurses that are seeking a relevant and robust measure to use in their unique practice setting.

## Additional files

Additional file 1: PRISMA-P 2015 Checklist. (DOCX 32 kb)

Additional file 2: Search strategy example MEDLINE. (DOCX 16 kb)

Additional file 3: Definitions of EIDM competence attributes. (DOCX 13 kb)

Additional file 4: Screening criteria. (DOCX 14 kb)

## Abbreviation

EIDM: Evidence-informed decision-making
