# Supplementary material for: Measures assessing attributes of evidence-informed decision-making (EIDM) competence among nurses: a systematic review protocol
Source: Syst Rev. 2018 Nov 3;7:181. doi: 10.1186/s13643-018-0849-8 (PMC6215345; doi:10.1186/s13643-018-0849-8)
Supplement: Supplementary file 2 — Search strategy example MEDLINE. (DOCX 16 kb) [file 13643_2018_849_MOESM2_ESM.docx]

**Additional file 2 Search Strategy Example MEDLINE**

Date limitations: 1990 – December 6, 2017

| 1. nurse*.mp. |
| --- |
| 2. exp Nurses/ |
| 3. 1 or 2 |
| 4. evidence based practice.mp. |
| 5. evidence informed decision making.mp. |
| 6. evidence based nursing.mp. |
| 7. Evidence-Based Practice/ |
| 8. Evidence-Based Nursing/ |
| 9. 4 or 5 or 6 or 7 or 8 |
| 10. measurement*.mp. |
| 11. assessment*.mp. |
| 12. psychometric*.mp. |
| 13. reliability*.mp. |
| 14. validity*.mp. |
| 15. questionnaire*.mp. |
| 16. survey*.mp. |
| 17. scale*.mp. |
| 18. tool*.mp. |
| 19. "Surveys and Questionnaires"/ |
| 20. Psychometrics/ |
| 21. 10 or 11 or 12 or 13 or 14 or 15 or 16 or 17 or 18 or 19 or 20 |
| 22. knowledge.mp. |
| 23. understanding.mp. |
| 24. comprehension.mp. |
| 25. Knowledge/ |
| 26. Comprehension/ |
| 27. 22 or 23 or 24 or 25 or 26 |
| 28. skill*.mp. |
| 29. "competency assessment".mp. |
| 30. 28 or 29 |
| 31. behavio?r*.mp. |
| 32. competenc*.mp. |
| 33. clinical decision making.mp. |
| 34. Professional Competence/ |
| 35. Clinical Competence/ |
| 36. Clinical Decision-Making/ |
| 37. 31 or 32 or 33 or 34 or 35 or 36 |
| 38. attitude*.mp. |
| 39. belief*.mp. |
| 40. professional value.mp. |
| 41. Attitude/ |
| 42. "Attitude of Health Personnel"/ |
| 43. 38 or 39 or 40 or 41 or 42 |
| 44. 3 and 9 and 21 |
| 45. 27 or 30 or 37 or 43 |
| 46. 44 and 45 |
