# Supplementary material for: Measures assessing attributes of evidence-informed decision-making (EIDM) competence among nurses: a systematic review protocol
Source: Syst Rev. 2018 Nov 3;7:181. doi: 10.1186/s13643-018-0849-8 (PMC6215345; doi:10.1186/s13643-018-0849-8)
Supplement: Supplementary file 3 — Definitions of EIDM competence attributes. (DOCX 13 kb) [file 13643_2018_849_MOESM3_ESM.docx]

**Additional file 3 Definitions of EIDM Competence Attributes**

|  | **Knowledge** | **Skills** | **Attitudes/Values** | **Behavioural** |
| --- | --- | --- | --- | --- |
| **Definition** | Understanding the defining theoretical, practical concepts and principles of EIDM and the different levels of evidence [31-34] | The application of EIDM knowledge to perform tasks related to EIDM in a practical setting [31-34] | Perceptions, personal beliefs about, and the importance assigned to EIDM  [31, 33] | The enactment of EIDM steps in a real-life health care setting  [31, 33, 35] |
| **Example** | Knowing the different steps of EIDM or the hierarchy of evidence. | The ability to apply research evidence to a clinical case scenario. | The belief that EIDM is associated with positive outcomes or the valuing each of the separate steps of the EIDM process. | Identifying a gap in patient care or critically appraising evidence related to a real world clinical practice question. |
