# Supplementary material for: Measures assessing attributes of evidence-informed decision-making (EIDM) competence among nurses: a systematic review protocol
Source: Syst Rev. 2018 Nov 3;7:181. doi: 10.1186/s13643-018-0849-8 (PMC6215345; doi:10.1186/s13643-018-0849-8)
Supplement: Supplementary file 4 — Screening criteria. (DOCX 14 kb) [file 13643_2018_849_MOESM4_ESM.docx]

**Additional file 4 Screening Criteria**

| **Title and Abstract Screening** |
| --- |
| 1) Does the study occur in a health care setting?  2) Does the study sample consist of nurses or a portion nurses?  **Notes:** (e.g., Registered Nurses, Licensed Practical Nurses, Public Health Nurses, Registered Practical Nurses)  3) Does the study involve a measure assessing any of the EIDM competence attributes (i.e., knowledge, skills, attitudes/values and/or behaviours)?  **Notes:**  **a) Knowledge:** an understanding of key theoretical and practical principles of EIDM, as well as the different levels of evidence  **b) Skills:** application of EIDM knowledge required to perform EIDM tasks such as developing a PICO question or critically appraising evidence  **c) Attitudes/Values:** perceptions, personal beliefs about, and the importance provided to EIDM  **d) Behaviour:** the practice of EIDM steps in a real world clinical setting |
| **Full Text Screening** |
| 1. Does the study occur in a health care setting? 2. Does the study consist of a quantitative design or mixed methods design? 3. Does the study sample consist of nurses or a portion of nurses? 4. If the study sample consists of a portion of nurses, is the data presented separately for nurses or can it be extracted separately? 5. Does the study involve a measure assessing any of the EIDM competence attributes (i.e., knowledge, skills, attitudes/values and/or behaviours)? 6. Is the study written in the English language?   **Notes:**  **a) Knowledge:** an understanding of key theoretical and practical principles of EIDM, as well as the different levels of evidence  **b) Skills:** application of EIDM knowledge required to perform EIDM tasks such as developing a PICO question or critically appraising evidence  **c) Attitudes/Values:** perceptions, personal beliefs about, and the importance provided to EIDM  **d) Behaviour:** the practice of EIDM steps in a real world clinical setting |
